# Vestibular and Oculomotor Findings in Vestibular Migraine Patients

**DOI:** 10.3390/audiolres13040053

**Published:** 2023-08-08

**Authors:** Sofia Waissbluth, Valeria Sepúlveda, Jai-Sen Leung, Javier Oyarzún

**Affiliations:** Department of Otolaryngology, Pontificia Universidad Católica de Chile, Santiago 8330033, Chile

**Keywords:** vestibular migraine, videonystagmography, vHIT, head impulse test, caloric response, nystagmus

## Abstract

Background: Vestibular migraine (VM) is the most frequent etiology of recurrent spontaneous episodic vertigo. Vestibular and oculomotor abnormalities have been described in VM; however, the diagnosis is currently based on symptoms. The objective of this study was to determine the most frequent abnormalities in videonystagmography (VNG), caloric testing (Cal) and video head impulse test (vHIT) in patients with VM. Methods: A retrospective cohort study was conducted, including all VM and probable VM patients seen from January 2021 to July 2022. Demographics, auditory symptoms and results via VNG, Cal and vHIT were evaluated. VNG results were compared with a control group. Results: Sixty patients, 81.7% with VM and 18.3% with probable vestibular migraine, were included. VNG revealed the following abnormalities: 21.7% spontaneous nystagmus; 33.3% positional nystagmus, mostly central; 26.7% optokinetic nystagmus; 56.7% smooth pursuit abnormalities and 70% saccade test abnormalities, mostly velocity and latency. An abnormal unilateral caloric response was seen in 22.9%, while vHIT revealed a low gain in at least one canal in 21.7%, and saccades were seen in at least one canal with normal gains in 18.3%. Concordant results between Cal and lateral vHIT were seen in 77.1% of cases. Conclusions: Although VM is a clinical diagnosis, vestibular and oculomotor abnormalities are commonly seen. The most frequent oculomotor findings were an abnormal saccade test, abnormal smooth pursuit and central positional nystagmus.

## 1. Introduction

Vertigo and migraine are frequent conditions affecting individuals throughout life [1]. According to estimates, approximately 15% of the general population experience migraines [2], and up to 15% of visits to general practitioners are due to dizziness [3]. Vertigo is two to three times more frequent in patients with migraines than in the general population [4,5]. Dieterich and Brandt first described vestibular migraine in 1999 as a migraine associated with episodic vertigo in patients who did not fulfill the criteria for basilar migraine established by the International Headache Society (IHS) [6]. In 2012, diagnostic criteria for vestibular migraine were published by the IHS–Bárány Society [7]. Nowadays, vestibular migraine is considered to be the most common cause of episodic vestibular syndrome worldwide, with a lifetime prevalence of 1–3%, and a 1-year prevalence of 0.9% [8]. Women are more frequently affected: 1.5 to 5 times more often than in men [4,6,9].

It has become apparent that a combination of genetic and environmental factors plays a role in causing vestibular migraine. In particular, a recent systematic review shed light on the genetic aspect of this condition, revealing a notable familial aggregation [10]. Although vestibular migraine can appear at any age, studies report a mean age of 37.7 years for the first occurrence in females, and 42.4 years in males [4,6,9,11], and it generally affects people with a prior history of migraines [4,12]. It is diagnosed with an average delay of 8.4 years after the first onset of migraine [13].

Vestibular function test results in vestibular migraine patients can be abnormal, particularly during or shortly after an episode [14,15], but they are not sufficiently specific to serve as diagnostic criteria [7]. To date, there have been no pathognomonic test results for vestibular migraine. Some non-specific vestibulo-ocular abnormalities have been reported in vestibular migraine patients, including spontaneous nystagmus, central positional nystagmus, gaze-evoked nystagmus and smooth pursuit abnormalities, amongst others, and they appear to increase over time [16]. Kang et al. reported that 11% of vestibular migraine patients exhibited abnormal video head impulse test (vHIT) results, and that 19% exhibited abnormal caloric test results [17]. In the symptom-free interval, the findings are generally normal, but unilateral peripheral vestibular signs such as canal paresis have been reported in 8 to 22%, and bilateral vestibulopathy has been reported in up to 11% [18]. Directional preponderance in the caloric test has also been described in approximately 8–15% [19,20,21]. 

The aim of the current study was to analyze oculomotor findings using videonystagmography (VNG), as well as vestibular function with vHIT and caloric testing, in a cohort of patients with vestibular migraine.

## 2. Materials and Methods

A retrospective cohort study was completed, including consecutive cases of vestibular migraine or probable vestibular migraine seen in the Pontificia Universidad Católica de Chile Healthcare Center from January 2021 to July 2022. Diagnosis was based on the consensus document of the Classification Committee of the Bárány Society [7]. Patients were excluded if they had concomitant Ménière’s disease, central pathology or if tests were not performed on the same day. During this time period, 109 cases were identified; however, seven were excluded because of concomitant Ménière’s disease, fifteen did not have VNG results, nine did not have vHIT results and eighteen did not have the tests performed on the same day. A total of 60 cases were finally included. Most cases were ictal when initially assessed. The VNG oculomotor testing, caloric test and vHIT results were analyzed. This study was approved by the local ethics committee of the Pontificia Universidad Católica de Chile. 

VNG was completed using the Interacoustics VisualEyes™ 525 system (Interacoustics; Middelfart, Denmark). Nystagmus was recorded and peak slow-phase velocity was documented. Spontaneous nystagmus, gaze-evoked nystagmus, positional nystagmus, bithermal caloric response, optokinetic nystagmus, smooth pursuit and saccade test were assessed. Bithermal caloric testing was performed with caloric stimuli consisting of alternate binaural irrigations with an air irrigator, with cold and hot temperatures (24 °C and 48 °C) for 1 min. Canal paresis was defined as a difference of ≥25% between both sides and was calculated using Jongkees’ formula. A control group was also tested using the same equipment; we included 30 consecutive patients that underwent VNG testing during a three-month period. The control group did not meet the criteria for vestibular migraine and did not have any central neurological symptoms or central causes of vertigo, neck or head injury. These patients were evaluated for tinnitus, sudden sensorineural hearing loss, benign paroxysmal positional vertigo (BPPV), anxiety disorders or acute vestibular vestibulopathy that they had recovered from (*n* = 30, 16 females, 52.8 ± 16.7 years of age (range 12–78)). None reported migraine headaches.

For vHIT testing, the right eye was recorded, and all three canals were evaluated (Otometrics ICS^®^ Impulse; GN Otometrics, Denmark). Subjects were fitted with the goggles, seated and asked to look at an eye-level target on the wall at a 1 m distance. Following calibration, the examiner, standing behind the patient, placed their hands on the participant’s head and performed repeated head impulses which were randomized in velocity and direction in the plane of the tested semicircular canal. Head impulses (150 to 300°/s) were continued until 20 head impulses were adequate (artifact-free) for each tested canal. All head impulses were completed by experienced practitioners. Parameters of abnormality were as follows: lateral canal VOR gain < 0.8, vertical canal VOR gain < 0.7 and/or presence of corrective saccades (covert and/or overt) in any canal. To compare caloric testing and vHIT results, we considered the vHIT to be abnormal if the lateral gain was <0.8, with or without refixation saccades present.

MRI (magnetic resonance imaging) of the brain was ordered for 40 patients, whereby all of which were normal.

Statistics: Descriptive statistics are presented as mean and standard deviation for continuous variables. The chi-square test was used to compare the rates of abnormal vestibular and oculomotor tests. A *p*-value of <0.05 was considered to be statistically significant.

## 3. Results

Sixty patients were included, with forty-nine females (81.7%), 45.0 ± 19.3 years of age (range of 9–79). Most met the criteria for vestibular migraine (81.7%), while a minority met the criteria for probable vestibular migraine (18.3%). Approximately half of the patients (51.7%) reported a history of symptoms greater than one year, while 48.3% reported symptoms less than one year. Auditory symptoms were commonly reported, including aural fullness, phonophobia and tinnitus (66.7%). Also, while accompanying headaches were described as one-sided, pulsating, moderate or severe and/or aggravated by routine physical activity, 38.3% of patients also reported headaches without these features. 

### 3.1. Videonystagmography

**Figure 1:**
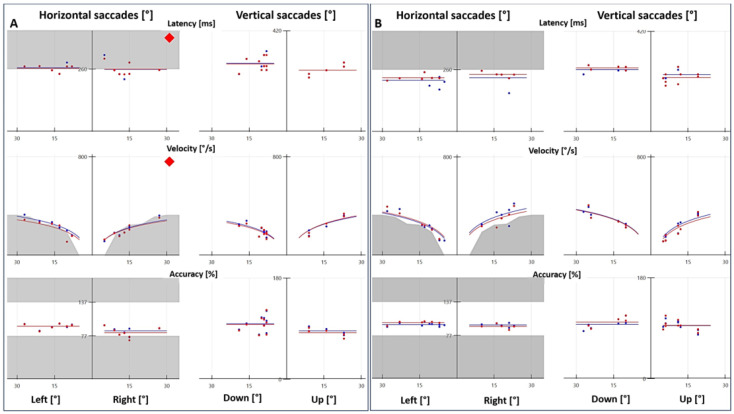
Saccade test with videonystagmography. (**A**) Patient with vestibular migraine presented abnormal latency and velocity, and the accuracy was within the normal range. (**B**) Age- and sex-matched control with normal latency, velocity and accuracy. The red and blue tracings represent the right and left eyes, respectively.

All of the patients completed the VNG test (Table 1); eight patients had a normal oculomotor VNG (excluding caloric testing). Spontaneous nystagmus was detected in 21.7%, most commonly first-degree nystagmus. Positional testing revealed central findings in the majority of cases (*n* = 17/20), and only three patients had BPPV for the posterior canal (one patient had both BPPV and central findings). The central findings (i.e., nystagmus that does not correspond to the plane being evaluated) included vertical nystagmus (eight upbeat), horizontal nystagmus (*n* = 9) and others (torsional, lateral and upbeat in different canals). The optokinetic nystagmus results were abnormal in 26.7% of cases, mostly in gain. The smooth pursuit was abnormal (saccadic pursuit, or slightly saccadic pursuit) in 56.7% of cases, and the saccade test was abnormal in 70% of cases (*n* = 42/60) (Figure 1). Abnormal velocity (*n* = 37/42) and the combination of abnormal velocity and latency (*n* = 19/42) were the most common findings. Accuracy was rarely affected. Finally, no patients presented gaze-evoked nystagmus. 

Because of the high rate of abnormal results in the saccade test, we decided to include a control group to compare VNG results (optokinetic nystagmus, smooth pursuit and saccade test). In the control group (*n* = 30), one patient had abnormal optokinetic nystagmus, two patients had abnormal smooth pursuit and two patients had an abnormal saccade test. These differences were statistically significant, with *p* = 0.0099, *p* < 0.00001 and *p* < 0.00001 for optokinetic nystagmus, smooth pursuit and saccade test, respectively.

### 3.2. Video Head Impulse Test

Results for the vHIT were normal (normal gains and absence of saccades) in 56.7% of the evaluated patients (*n* = 34). A low gain in at least one canal was observed in 21.7% (*n* = 13), while saccades in at least one canal with a low gain in at least one canal were seen in six patients (11.7%). Interestingly, we observed the presence of saccades in at least one canal with normal gains in 11 cases (18.3%). Of the thirteen patients with spontaneous nystagmus, six had a normal vHIT, five had saccades in the horizontal canal (mostly bilateral) and two had a low gain and saccades in the horizontal canal.

**Figure 2:**
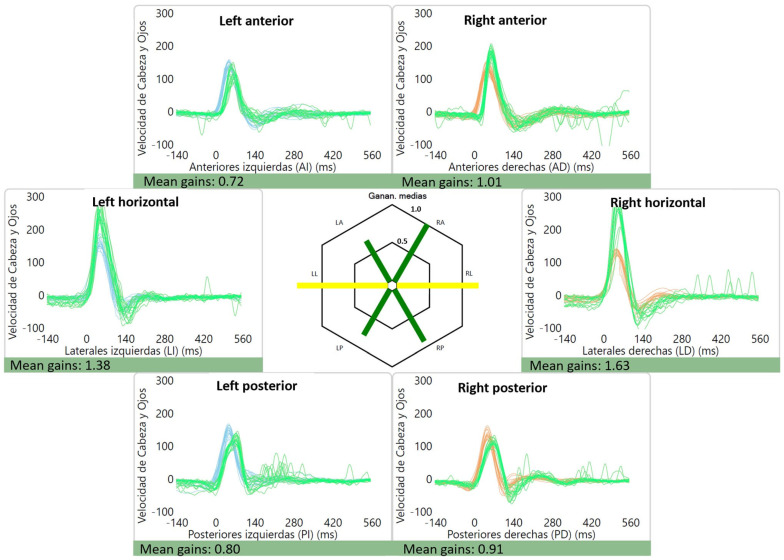
Abnormal lateral canal vHIT gains for a patient with vestibular migraine. High gains can be seen for the lateral canals: 1.38 for the left lateral canal and 1.63 for the right lateral canal. Gains for the vertical canals were within the normal range. The green line represents the “eye data”, i.e., the eye movement during and after the impulse (vestibulo-ocular reflex) while the blue and orange represent “head data” for the left and right side, respectively.

**Figure 3:**
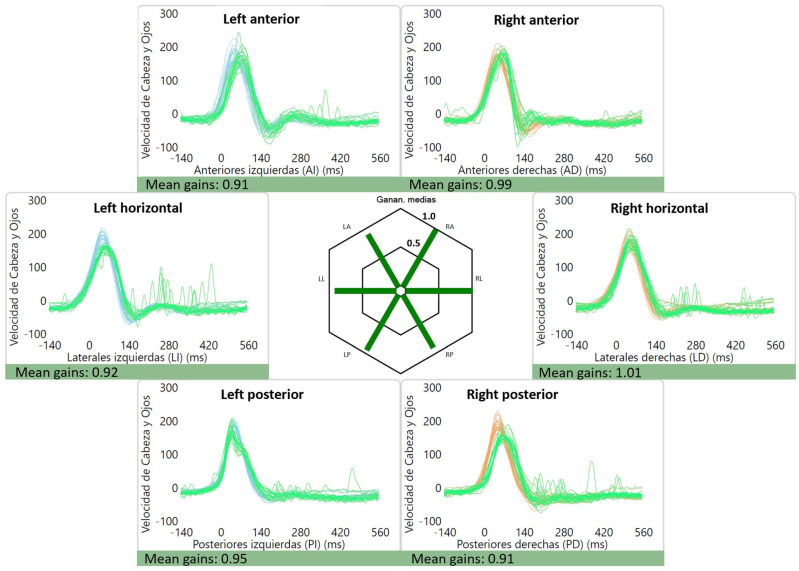
Normal vHIT gains and absence of saccades for a patient with vestibular migraine.

The average gains were as follows: right lateral canal: 1.01 ± 0.12, left lateral canal: 0.93 ± 0.11, right anterior canal: 0.90 ± 0.11, left anterior canal: 0.86 ± 0.10, right posterior canal: 0.84 ± 0.10 and left posterior canal: 0.80 ± 0.12. Of interest, two patients had high gains (≥1.20) for the lateral canals, which were bilateral in one case (1.38 and 1.63) (Figure 2) and unilateral in the other (1.27). An example of a normal vHIT is shown in Figure 3.

### 3.3. vHIT and Caloric Testing

Not all of the patients had a caloric test performed; 35 patients had both caloric and vHIT tests performed on the same day. Most cases had a normal caloric response (77.1%, 27/35), while eight had unilateral hypofunction with an average weakness of 38% (range of 26–56%). Hence, 77.1% of cases had concordant results between the caloric response and lateral canal gain via vHIT (Table 2). No directional preponderance was detected for this cohort. For the patients that had all three tests (VNG, caloric and vHIT), only two patients had normal test results. 

### 3.4. Abnormalities over Time

A summary of the abnormal test results can be seen in Table 3. Because there is evidence that oculomotor and vestibular findings in vestibular migraine patients change over time [22], we also analyzed the abnormal results between patients reporting symptoms of less than one year (*n* = 29) vs. greater than one year (*n* = 31; average of 4.9 years, range of 1–20 years). We did not detect any significant changes between the two groups.

## 4. Discussion

While vestibular migraine is a clinical diagnosis and there are no pathognomonic vestibular findings, oculomotor and vestibular abnormalities are not uncommon. In our cohort of patients with vestibular migraine and probable vestibular migraine, only 2 out of 35 patients had a normal VNG, caloric response and vHIT. Overall, seven patients (7/60) had a normal VNG (oculomotor testing), 56.7% (34/60) had a normal vHIT and 77.1% (27/35) had a normal caloric response. The most frequent oculomotor findings were as follows: abnormal saccade test, abnormal smooth pursuit and central positional nystagmus.

Abnormal test results are frequently observed during attacks, i.e., ictal signs and symptoms. However, abnormal interictal neuro-otologic findings are common [23]. Most patients in this cohort were ictal at presentation; nevertheless, vestibular migraine patients can have recurrent symptoms that vary in intensity, and it is somewhat difficult, for certain patients, to determine ictal vs. interictal when being assessed.

Our results for caloric response are consistent with the recent literature. Unilateral caloric weakness was observed in 22.9% of cases. For instance, Yilmaz et al. observed caloric weakness in 34% and abnormal vHITs in 18% of vestibular migraine patients during interictal assessment [19], while Li et al. described caloric weakness in 19.4% for acute episodes [24]. Caloric weakness is variable and has been described in up to 56% of patients with vestibular migraine [17,20,22,25,26,27,28,29,30]. 

Abnormal results for the vHIT are also variable because there are many types of abnormalities that can be described: low gains and/or saccades. With our criteria for abnormality (low gain in any canal and/or saccades in any canal), 43.3% of vestibular migraine patients had at least one abnormality. The most frequent findings were a low gain in at least one canal (21.7%) and the presence of saccades in at least one canal with normal gains (18.39%). Elsherif et al. observed normal gains with saccades in 36% of migraineurs [31]. In another study, they reported that 26% of vestibular migraine patients exhibited abnormal vHIT results: saccades with normal gains in 18%, and low gains with saccades in 7.5% [31]. Fu et al. described abnormal lateral canal vHIT in 15%, and vertical canal vHIT in 27% of patients with vestibular migraine [25]. 

With regard to oculomotor findings, 88.3% of the patients in this cohort had at least one abnormal finding on VNG testing, most commonly latency and/or velocity for the saccade test, followed by saccadic/mildly saccadic smooth pursuit and central positional nystagmus. Our prevalence of saccade test abnormalities (70%) is greater than that previously described in other studies, which vary from 0 to 32% [20,21,22,24,27,31,32]. The reasons for such a discrepancy could be due to different VNG equipment, ictal vs. interictal testing and the degree of what is considered to be abnormal. Smooth pursuit abnormalities were seen in 56.7%; this is within the reported range in vestibular migraine patients, which has been described to be between 0 and 85.4% [20,21,22,24,25,27,31,32]. Central positional nystagmus was also common, seen in 30% of vestibular migraine patients (*n* = 18/60). This is also a commonly reported finding in vestibular migraine patients [20,21,22,23,25,26,27,31,32]. No patient had gaze-evoked nystagmus and 21.7% had spontaneous nystagmus. 

The pathophysiology for vestibular migraine remains unclear but there seems to be multiple factors that flow into abnormalities of the central nervous system and of inner ear potassium homeostasis [33]. Some authors suggest a familial aggregation of patients with migraine because approximately 40 to 90% of patients have a positive family history [33]. Some genes such as CACNA1A, NOTCH3, TREX1 and COL4A1 are associated with nystagmus or vasculopathies related to migraines. Baloh [33] suggests that these genes are related to ion channel alterations which produce a local buildup of potassium in the extracellular space, with a cortical depression wave spreading. This phenomenon is associated with hypersensitivity of the trigeminal fibers surrounding pial arteries. These fibers release substance P and calcitonin gene-related peptide (CGRP), resulting in vascular permeability, dilatation of cerebral vessels and a local inflammatory response, vasospasm and activation of pain-provoking fibers of the trigeminal vascular system. Thus, acute episodes of hearing loss and/or vertigo associated with migraine could be explained by vasospasm of the cochlear and/or vestibular branches of the internal auditory artery [33].

Recently, neuroimaging has shed some light on structural and functional brain alterations in patients with vestibular migraine. During attacks, it appears that the vestibulo–thalamo–cortical pathway is activated, and that the metabolism in the occipital cortex is decreased as measured by (18)F-fluorodeoxy glucose positron emission tomography [34,35,36]. Shin et al. suggest that this may indicate reciprocal inhibition between the visual and vestibular systems [37]. Other findings include an abnormal thalamic functional response following vestibular stimulation (i.e., ear irrigation with cold water) seen with whole-brain blood-oxygen-level-dependent (BOLD) functional MRI [38]. Grey matter volume changes have also been observed in vestibular migraine patients, with a volume increase in the frontal and occipital regions and in the left thalamus. Interestingly, these grey matter abnormalities were not correlated with disease duration and attack frequency [34]. Resting-state functional MRI and three-dimensional T1-weighed MRI have also shown reduced grey matter volume in the bilateral parietoinsular vestibular cortex, right middle frontal gyrus and precuneus grey matter [35]. In contrast, Wang et al. [36] reported an increase in grey matter volume in the bilateral superior frontal gyrus and the right angular gyrus, which might be related to self-adaptation of the nervous system, leading to abnormal brain sensitization.

The diagnosis of vestibular migraine and probable vestibular migraine is clinical; however, oculomotor and vestibular findings are not uncommon, especially during an attack. Because none of these findings are pathognomonic, they cannot be used as diagnostic criteria. However, these tests can be useful in the differential diagnosis of recurrent vestibulopathy. Recently, an interesting ocular finding has been described for vestibular migraine patients. Gufoni and Casani evaluated the pupillary hippus in vestibular migraine patients, where the pupils dilate and contract bilaterally and cyclically in the presence of constant lighting, independently of light intensity. It is a non-specific sign and can be seen physiologically during a drowsy state. They reported that 28 out of 30 patients with vestibular migraine exhibited pupillary hippus, or “dancing pupils” [39]. It is worth noting that this sign was observed during the inter-critical phase. This phenomenon is believed to arise from a dysregulation of the central parasympathetic nervous system activity [40]. Gufoni and Casani recommend looking for pupillary hippus when evaluating vestibular migraine patients; hence, this could be considered an added test in oculomotor evaluation when assessing these patients [39].

Recently, other less frequently described findings have been reported for patients with vestibular migraine, including opsoclonus and direction-changing spontaneous nystagmus. Lee et al. retrospectively reviewed over four thousand cases seen at their dizziness clinic from 2013 to 2020. Of the 4786 cases, 41 exhibited direction-changing spontaneous nystagmus, and of these, two had vestibular migraine. The nystagmus had slow-phase velocity that was weak, around 1–2°/s, and was seen during the interictal period. They also reported that hyperventilation-induced nystagmus and head-shaking nystagmus were not direction-changing in either patient [41]. They could not establish periodicity because it was a retrospective study, and not all of the recordings were standardized for a certain duration of time; hence, they could not establish whether it was periodic or aperiodic direction-changing spontaneous nystagmus that was actually recorded. Also, they did mention variability as to when vestibular function tests were performed with regard to the onset of symptoms. And, finally, the question remains as to whether these findings are clinically relevant or not [41]. Very recently, Kim et al. published two cases of opsoclonus induced by horizontal head-shaking in vestibular migraine patients. These patients met the diagnostic criteria proposed by the Bárány Society for vestibular migraine and did not exhibit any other brainstem or cerebellar dysfunctions [42]. Opsoclonus is an involuntary eye movement disorder characterized by rapid and repetitive conjugate eye movements that do not follow any particular pattern, and do not present intersaccadic intervals. It has mostly been associated with paraneoplastic and infectious disorders, but also toxic and metabolic disorders, as well as being drug-induced [43]. Characteristics that were seen in these two patients with vestibular migraine were as follows: 1. they did not exhibit any other brainstem or cerebellar syndromes, 2. opsoclonus only appeared following head-shaking (or positioning without visual fixation), 3. opsoclonus had a rhythmicity of 5–6 Hz and, 4. it lasted for several minutes after the head-shaking maneuver [42]. They suggested that this type of opsoclonus in certain patients with vestibular migraine could be a result of unstable or hyperactive neural circuits between the excitatory and inhibitory saccadic premotor burst neurons [42].

Some authors have aimed to assess whether certain vestibular abnormalities in vestibular migraine patients are associated with response to treatment. For instance, Jung et al. described that indicators of a poor prognosis in vestibular migraine patients who experienced recurrent attacks for over six months were abnormal vestibular ratios on posturography, and abnormal vestibular-evoked myogenic potential (VEMP) responses. Interestingly, 61% of patients with a normal VEMP exhibited complete remission from headaches, whereas only 30% of patients with an abnormal VEMP experienced such remission. In addition, there was no noteworthy association between abnormal caloric results and treatment response, although there was a tendency toward a poor response for vertigo, not for headaches [44]. On the other hand, Kang et al. reported that at a six-month follow-up, there was a significant correlation between a need for ongoing medication and abnormal vHIT gain as well as abnormal caloric results in a cohort of vestibular migraine patients. Furthermore, neither cervical VEMP nor ocular VEMP responses could predict the drug response in this cohort [17]. Further prospective research is needed in order to better understand vestibular and oculomotor findings in vestibular migraine patients, its pathophysiology and response to treatment.

Another interesting aspect assessed by previous authors is the presence of vestibular and oculomotor abnormalities not only in vestibular migraine patients but also in patients with migraine without vertigo. Casani et al. completed a prospective observational study comparing migrainous patients with and without vestibular symptoms. They were assessed during the interictal phase, and they reported neurotologic abnormalities in 36.3% and 31.8%, respectively. The incidence of abnormalities was similar between both of the groups, as well as central vestibular involvement. The most common findings in both of the groups included bilateral hyperresponsiveness and canal paresis. Remarkably, both of the groups had patients with eye tracking test abnormalities (saccadic pursuit and saccadic inaccuracy) and positional nystagmus of the central type. The authors concluded that migraine can affect the vestibular pathways, even when these migrainous patients did not report vestibular symptoms [45]. Boldingh et al. also compared interictal vestibular abnormalities in patients with vestibular migraine and patients with migraine without vertigo. The vestibular test battery was extensive and included fourteen tests: seven were evaluated with videonystagmography and seven were evaluated at bedside. They reported that 70% of patients with vestibular migraine and 38% of patients with migraine without vertigo had pathological findings in one or more of the 14 tests. Both had similar rates of abnormal positioning nystagmus and unilateral caloric paresis. The tests that showed statistically significant differences between both of the groups were Romberg’s test, impaired visual fixation suppression of the VOR and positional test (static). Abnormalities in these tests were more common in vestibular migraine patients. Also, although the difference did not reach statistical significance, the smooth pursuit test by videonystagmography was abnormal in 13.2% of patients with vestibular migraine [20]. The authors suggested that subclinical vestibular dysfunction may be part of migraine pathology in general. 

The limitations of this study include the fact that not all of the patients underwent caloric testing, and no otolithic function testing and posturography were performed. We did not assess pupillary hippus, as this study was completed before the above-mentioned publication by Gufoni and Casani. Also, we must consider the intrinsic limitations of a retrospective study, where certain details of the history could be lacking. This study’s clinical significance lies in demonstrating the prevalence of vestibular and oculomotor findings in vestibular migraine patients, particularly when examined during the ictal phase.

## 5. Conclusions

In a cohort of vestibular migraine patients, the most frequently observed oculomotor findings on VNG were an abnormal saccade test, abnormal smooth pursuit and central positional nystagmus. As for the vestibular tests, unilateral caloric weakness was more commonly observed as compared to vHIT abnormalities; 22.9% had discordant results. Some vestibular migraine patients exhibit saccades with normal gains on vHIT. No significant differences were seen in VNG when comparing patients with symptoms lasting for less than a year and greater than one year. While vestibular migraine is a clinical diagnosis, vestibular and oculomotor abnormalities are commonly seen. Further research is needed to understand the pathophysiology of this condition and its related oculomotor findings.

## Figures and Tables

**Table 1:** Videonystagmography abnormal results—oculomotor testing.

Test	*n*	%	Specific Findings (*n*)
Spontaneous nystagmus	13	21.7%	First degree: 10 Second degree: 2 Third degree: 1
Positional nystagmus	20	33.3%	Central findings: 17 BPPV only: 2 Central findings and BPPV: 1
Optokinetic nystagmus	16	26.7%	Gain only: 13 Symmetry only: 1 Gain and symmetry: 2
Smooth pursuit	34	56.7%	-
Saccade test	42	70%	Overall: latency: 23, accuracy: 1, velocity: 37 By parameter: Latency only: 3 Accuracy only: 0 Velocity only: 16 Combinations: Latency and velocity: 19 Latency, accuracy and velocity: 2
Gaze-evoked nystagmus	0	0	-

**Table 2:** vHIT and caloric testing in vestibular migraine patients.

	Caloric Response—Normal	Caloric Response—Unilateral Weakness
vHIT—normal	26	7
vHIT—abnormal *	1	1

* Low gain for the lateral canal, with or without saccades for that canal.

**Table 3:** Abnormalities over time for vestibular migraine.

Abnormal Test Results	Less than One Year (*n* = 29)	Over One Year (*n* = 31)	*p*-Value
vHIT	34.5%	48.4%	0.2749
Caloric response	20.7%	12.9%	0.1571
Spontaneous nystagmus	24.1%	19.3%	0.6531
Positional nystagmus *	24.1%	32.3%	0.4854
Optokinetic nystagmus	24.1%	29%	0.6683
Smooth pursuit	55.2%	58%	0.8212
Saccade test	68.9%	70.1%	0.3497

*p*-value by hi-square test. * Excluding BPPV, central findings only.

## Data Availability

Not applicable.
